
JOURNAL INFORMATION
==============================
NLM Title Abbreviation: J High Energy Phys
Journal ID: J High Energy Phys
NLM Journal ID: 101668727

Journal of High Energy Physics

ISSN: 1126-6708
EISSN: 1029-8479

ARTICLE INFORMATION
==============================
PMCID: PMC8827299
PMID: 35226713
DOI: 10.1007/JHEP03(2016)076
Article ID: 3337
Article version: 1
Subjects: Erratum

Erratum to: Three-dimensional fractional-spin gravity

Boulanger Nicolas nicolasboul@gmail.com
1
Sundell Per per.anders.sundell@gmail.com
2
Valenzuela Mauricio valenzuela.u@gmail.com
1 3
1 grid.8364.9 000000012184581X Service de Mécanique et Gravitation, Université de Mons - UMONS, Mons, Belgique
2 grid.412848.3 000000012156804X Departamento de Ciencias Físicas, Universidad Andres Bello, Republica 220, Santiago, Chile
3 grid.7119.e 000000040487459X Instituto de Ciencias Físicas y Matemáticas, Universidad Austral de Chile - UACH, Valdivia, Chile
Electronic publication date: 2016 Mar 14
Print publication date: 2016
Volume: 2016
Issue: 3
Electronic Location ID: 76
Received 2016 Mar 2; Accepted 2016 Mar 3
Copyright: © The Author(s) 2016
License: Open AccessThis article is distributed under the terms of the Creative Commons Attribution 4.0 International License (https://creativecommons.org/licenses/by/4.0), which permits use, duplication, adaptation, distribution, and reproduction in any medium or format, as long as you give appropriate credit to the original author(s) and the source, provide a link to the Creative Commons license, and indicate if changes were made.
License URL: https://creativecommons.org/licenses/by/4.0/

Erratum for: 10.1007/JHEP02(2014)052
issue-copyright-statement: © SISSA, Trieste, Italy 2016

==============================
Open Access

This article is distributed under the terms of the Creative Commons Attribution License (CC-BY 4.0), which permits any use, distribution and reproduction in any medium, provided the original author(s) and source are credited.

Erratum to: JHEP02(2014)052

ArXiv ePrint: 1312.5700

Associate researcher of the FNRS, Belgium (Nicolas Boulanger).

